# The use of denosumab in rare bone diseases in adults: a systematic review from the ECTS Rare Bone Disease Action Group

**DOI:** 10.1210/clinem/dgag154

**Published:** 2026-04-07

**Authors:** Oana O Bulaicon, Femke M van Haalen, Gavin P R Clunie, Corinna Grasemann, Willem F Lems, Polyzois Makras, Geert Mortier, Ralf Oheim, Adalbert Raimann, Heide Siggelkow, Gaia Tabacco, Robert J P van der Wal, Pieter B de Witte, Natasha M Appelman-Dijkstra

**Affiliations:** Center for Bone Quality, Leiden University Medical Center, 2333 ZA Leiden, the Netherlands; Center for Bone Quality, Leiden University Medical Center, 2333 ZA Leiden, the Netherlands; Metabolic Bone Physician, Cambridge University Hospitals, Box 204, Hills Rd, Cambridge CB2 0QQ, UK; Department of Pediatrics, Katholisches Klinikum Bochum, Ruhr-University Bochum, Bochum 44791, Germany; Department of Rheumatology, Amsterdam University Medical Center, 1105 AZ Amsterdam, the Netherlands; LCH Adult Clinic, Department of Medical Research, and Department of Endocrinology and Diabetes, 251 Hellenic Air Force and VA General Hospital, Athens 11525, Greece; Center for Human Genetics, University Hospital Leuven and KU Leuven, Leuven B-3000, Belgium; Department of Osteology and Biomechanics, University Medical Center Hamburg-Eppendorf, Hamburg 22529, Germany; Department of Pediatrics & Adolescent Medicine, Vienna Bone and Growth Center, Medical University Vienna, Vienna 1090, Austria; Clinic of Gastroenterology, Gastrointestinal Oncology and Endocrinology, University Medical Center Göttingen, Göttingen 37075, Germany; MVZ Endokrinologikum Göttingen, Göttingen 37075, Germany; Unit of Metabolic Bone and Thyroid Disorders, Fondazione Policlinico Universitario Campus Bio-Medico, Rome 00128, Italy; Department for the Promotion of Human Sciences and Quality of Life, San Raffaele Open University, Rome 00166, Italy; Department of Orthopaedics, Leiden University Medical Center, 2333 ZA Leiden, the Netherlands; Department of Orthopaedics, Leiden University Medical Center, 2333 ZA Leiden, the Netherlands; Center for Bone Quality, Leiden University Medical Center, 2333 ZA Leiden, the Netherlands

**Keywords:** RANKL, denosumab, rare bone diseases, fibrous dysplasia, McCune-Albright syndrome, aneurysmal bone cyst, cherubism

## Abstract

**Context:**

Rare bone diseases (RBDs) may display a disrupted RANKL-RANK-osteoprotegerin pathway causing increased osteoclastogenesis and enhanced bone resorption. Although bisphosphonates are commonly used, they often fall short of desired outcomes. Denosumab, an anti-RANKL antibody, provides a promising alternative by swiftly and strongly suppressing bone turnover (faster and more potent suppression of bone resorption than bisphosphonates), though its effects are reversible on discontinuation. The use of denosumab has been highlighted, especially in pediatric cases, but not substantially in adults.

**Evidence Acquisition:**

A targeted evidence search was conducted to retrieve studies reporting denosumab use in RBDs in adults.

**Evidence Synthesis:**

Denosumab administration may lead to pain reduction, lesion reduction, or bone formation. Treatment dosage, schedules, and duration varied, however, a dose of 120 mg dosed monthly or every 3 months for almost 1 year reached the desired treatment effect in most patients. Denosumab is generally well tolerated in adults, with mild common side effects such as (asymptomatic) hypocalcemia and hypophosphatemia. Serious adverse effects such as osteonecrosis of the jaw or atypical femoral fractures are rarely reported. Main concerns regard rebound effect after denosumab discontinuation, with disease recurrence in some cases. Zoledronic acid after discontinuation of denosumab might be advisable, but is seldom reported.

**Conclusion:**

Denosumab is a feasible treatment in adults with RBDs when managed by multidisciplinary teams with knowledge of both the underlying disease and potential surgeries as well as the medical site of treatment. Denosumab discontinuation management is paramount to prevent recurrence and severe complications. The paucity of data supports the need for data collection through rare disease registries for future pertinent evidence-based recommendations.

The RANKL-RANK-osteoprotegerin (OPG) pathway plays a crucial role in regulating bone resorption and formation, determining bone turnover status (1). Disruptions in this pathway with enhanced osteoclastogenesis have been observed in rare bone diseases (RBDs) (2-4). For example, there may be increased RANKL production due to mutated osteoblasts (fibrous dysplasia/McCune-Albright syndrome [FD/MAS]) (5, 6). Another option might be that there is an increased osteoclastogenesis with the production of giant osteoclasts (OCs) with secondarily increased bone resorption such as in aneurysmal bone cysts (ABCs) (7), Langerhans cell histiocytosis (LCH) (8), or cherubism (9). This increased osteoclastogenesis may be present due to increased RANK-RANKL activity, like in Hajdu-Cheney syndrome (HCS), in which it is caused by a *NOTCH2* pathogenic variant, or in familial expansile osteolysis caused by pathogenic variants in tumor necrosis factor superfamily member 11 (*TNFRSF11*) (4), while in some diseases, like Gorham-Stout disease (GSD), a precise etiology has not yet been identified (10). Although different mechanisms are involved, all these diseases lead to clinical impairment associated with pain, deformities, fractures, or even malignancies. In most RBDs, there is no effective treatment or cure. Tackling the RANK-RANKL pathway, therefore, may offer new possible treatment options, especially in the form of the antireceptor activator of the nuclear κ-B ligand (RANKL) antibody denosumab. The use of denosumab has recently been reviewed in children with RBDs (11), but is an attractive potential treatment in adults as well, since invasive procedures like surgery have their limitations due to the extent of the disease, lesion location (eg, spine/pelvis), patient comorbidities, risk of local recurrence, morbidity of resection, and the lack of reconstructive options. Altering bone turnover has been tried with bisphosphonates for decades as they have been an alternative in high-turnover diseases like Paget disease, reducing pain, and preventing further bone involvement/damage (12). However, the use of bisphosphonates in many RBDs has not been shown to be effective in decreasing disease activity, lesion size, or correcting deformities (13, 14). Potentially, denosumab may offer a faster and more effective suppression of bone turnover, despite the fact that its effects are reversible on treatment discontinuation (15). This regimen includes an initial *“loading dose”* phase, a term derived from oncology practice describing more frequent administration at treatment initiation, specifically, denosumab 120 mg, given at the start of treatment with loading of 120 mg on days 1, 8, 15, and 29, followed by a monthly schedule of 120 mg every 4 weeks. This schedule is currently approved as medical treatment for patients with advanced or inoperable giant cell tumor of the bone (GCTB). The approval of denosumab for GCTB was based on 2 open-label, single-arm studies involving 548 patients. Notably, no evidence supports the indication of the loading doses; however, this is the commonly used treatment schedule (16-18).

Denosumab discontinuation management is particularly important due to additional risks. Across the published literature on osteoporosis, denosumab discontinuation confers the risk of rebound high bone turnover occasionally with associated hypercalcemia and increased risk of vertebral fragility fractures (19, 20).

The European Calcified Tissue Society (ECTS), through its Rare Bone Diseases Action Group, develops guidelines and position papers that offer science-based information to support stakeholders in their clinical and research practice (www.ects.com).

Accordingly, our objective was to review the medical literature to summarize current knowledge on the use of systemic off-label denosumab in adults with RBDs, and identify existing knowledge gaps and priorities for future research.

## Methods

This systematic review was conducted according to the Preferred Reporting Items for Systematic Reviews and Meta-Analysis (PRISMA) guidelines (Supplemental material 1) (21) and was not registered on Prospero. The evidence search was performed in collaboration with a trained medical librarian with extensive experience in systematic literature reviews and evidence-based search strategies (November 1, 2024, search provided in Supplemental material 2: PubMed, Embase, Web of Science) (21).

**Figure 1:**
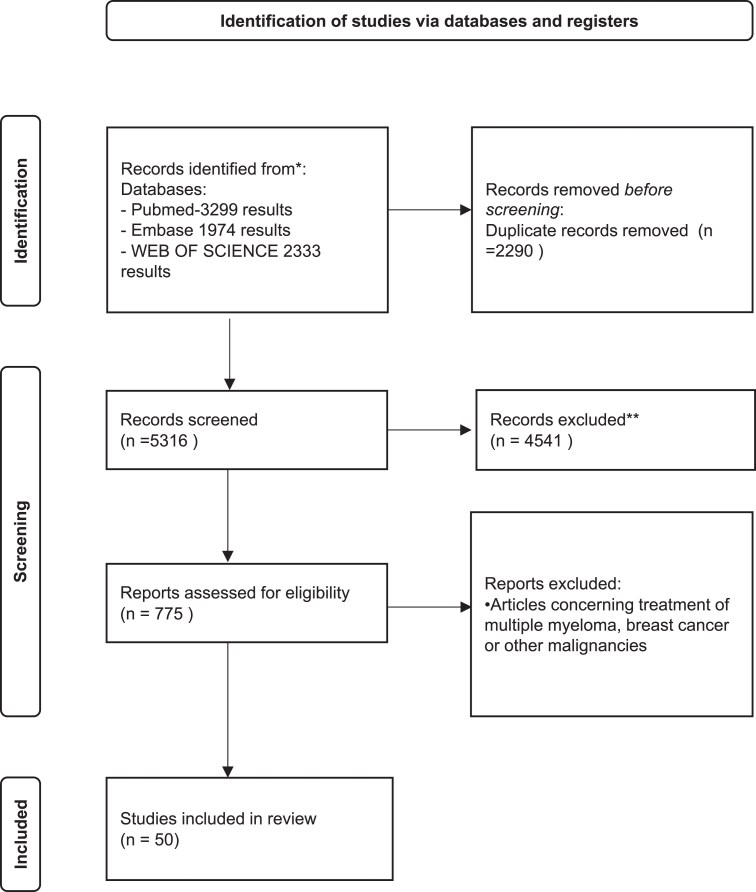
Results literature research—PRISMA flow diagram. ** Exclusion criteria: - Title/Abstract not in the English language. - Article describing pediatric cases. - Article not including the list of diseases to be searched.

One author (O.O.B.) performed the preliminary screening of all retrieved studies based on their titles and abstracts, which was checked by N.A.D. Articles were included if they reported RBDs with increased osteoclast activity via the RANKL-RANK-OPG pathway and treatment with systemic denosumab. We excluded papers on children as they were extensively reviewed by Vanderniet and colleagues (11). With regard to rare diseases, evidence is limited, thus we decided to also include case reports and small case series. (Supplemental material 3; Fig. 1) (21). Relevant reviews were retrieved to form an overview of the current status. Data regarding disease details, treatment indication, clinical, paraclinical effects, side effects, and discontinuation strategy were retrieved. Data were summarized in disease-specific tables using a uniform format, with outcomes reported according to the original authors' descriptions. Due to the nature of the available evidence, outcome definitions and measurement methods varied across studies. (Supplementary material 3) (Table 1) (21) We included diseases reviewed in children (11) and extended it to other rare bone conditions that are currently treated with denosumab by the authors of the paper. We excluded papers on diseases characterized by low bone mass, such as osteogenesis imperfecta. Pediatric literature was referenced only to provide mechanistic insights, particularly regarding drug discontinuation and rebound effects, and was not used to inform adult dosing or clinical recommendations.

**Table 1:** Denosumab in adults with rare bone diseases (treatment details from existing evidence)

Indication	Dose and regimen (most used)	Treatment duration	Clinical results	Radiologic results	Surgery downstage	Recurrence	Side effects	Exit strategy data available	No. of patients
**ABCs**	120 mg monthly with loading doses (days 8 and 15)	1 y	Pain reduction	Lesion reduction, bone formation	Yes	No, majority of patients	No	No	22
	60 mg monthly	9 mo	Functional improvement	Lesion reduction, bone formation	Yes	No, majority of patients	No	No	20
**CGCG**	120 mg monthly with loading doses days 8 and 15	6 mo	Pain reduction	Lesion reduction, bone formation	—	No	No	No	17
	120 mg monthly	9-12 mo	Pain reduction	Lesion reduction, bone formation	—	No, majority of patients Yes	No, majority of patients Hypocalcemia	No	12
	60 mg every 3 mo	1 y	Pain reduction	Lesion reduction, bone formation	—	—	No	No	2
**Cherubism**	120 mg monthly with loading doses days 8 and 15	6 mo	—	Bone formation	—	—	No	No	1
	60 mg every 6 m	2.5 y	Pain reduction, functional improvement	Lesion reduction, bone formation	Yes	No	No	No	1
**FD/MAS**	120 mg with loading doses, then monthly/bimonthly	6-12 mo	Pain reduction	Bone formation	—	Bone turnover markers rebound	Hypercalcemia after denosumab discontinuation	Yes, zoledronic acid at different time points	11
	120 mg every 6 mo	1 y	Pain reduction	—	—	Bone turnover markers rebound	Asymptomatic hypocalcemia and/or hypophosphatemia	Yes, zoledronic acid at different time points	4
	60 mg every 3-6 mo	1-3 y	Pain reduction	Bone formation	—	Bone turnover markers rebound	Asymptomatic hypocalcemia and/or hypophosphatemia; mild asymptomatic hypercalcemia at discontinuation	Yes, zoledronic acid at different time points	66
**GSD**	60 mg every 6 mo	—	Pain reduction Clinical improvement	Lesion reduction; bone formation	—	—	No	No	3
**HCS**	60 mg every 6 mo	—	Pain reduction	Stopped/no effect acro-osteolysis	—	—	No	No	2
**LCH**	120 mg every 2 mo (4 consecutive doses)	8 mo	Pain reduction	Lesion reduction; bone formation	—	No	No	No	12

**Abbreviations:** ABCS, aneurysmal bone cysts; CGCG, central giant cell granulomas; FD/MAS, fibrous dysplasia/McCune-Albright syndrome; GSD, Gorham-Stout disease; HCS, Hajdu-Cheney syndrome; LCH, Langerhans cell histiocytosis.

In this manuscript, denosumab dosing is described according to the administered dose and indication. The 60-mg dose refers to the osteoporosis regimen, typically administered every 6 months, but in some reported cases administration was every 3 months. The 120-mg dose refers to the oncology regimen, typically administered every 4 weeks with additional loading doses. The term *“maintenance”* refers to continued treatment after initial disease control, usually with extended dosing intervals or dose adjustments.

Risk of bias was assessed using the Joanna Briggs Institute (JBI) critical appraisal checklists for case reports and case series (Supplemental material 4) (21)

## Results

### Literature search

The literature search resulted in 5316 papers. Publications in the English language that addressed the medical treatment of the diseases with denosumab based on title and abstract were selected for full text review. After full text review, 47 papers fulfilled the inclusion criteria and were retrieved.

### Aneurysmal bone cysts

#### Disease background

An ABC (OMIM No. 602929) is a rare, locally aggressive bone lesion (22), characterized by expansile cystic lesions filled with blood and OCs like giant cells (7).

ABCs occur as primary lesions in most cases or may be secondary to other bone diseases, such as FD/MAS, GCTB, osteoblastoma, chondroblastoma, or low-grade sarcoma. ABCs most commonly occur in children, but are reported in adults as well (23, 24).

The etiopathogenesis of ABCs is unknown, but it is assumed that chromosomal rearrangements involving the *USP6* gene trigger lesion development (25). ABCs occur most frequently in vertebral bodies, the pelvis, and metaphyses of long bones (24). ABCs typically cause pain, bone deformity, impaired musculoskeletal function, bone destruction, and increased fracture risk at the site of the lesion (24).

Currently, several treatment strategies are used in clinical practice, including watchful waiting, sclerotherapy, embolization, intralesional (ie, curettage), or en bloc surgical resection (24, 26). However, ABCs have a high risk of recurrence after current treatment strategies (26).

#### Treatment with denosumab

Denosumab treatment of ABCs has been reported as either a primary treatment, secondary treatment after failure of other therapy, such as recurrence or insufficient pain control, or as adjuvant therapy to surgery in 42 patients, including both surgical and nonsurgical situations (27). Denosumab treatment is associated with reduction of both pain and ABC size and increased lesion mineralization (28-32). Where the site of an ABC makes surgery impossible, risky, or challenging, medical therapy such as denosumab might be a relevant alternative, with several reports highlighting the positive outcomes, such as reducing pain or ABC size (33-35).

Optimal denosumab doses in treating ABCs are not known but in reported cases, denosumab has been given as 120 mg monthly with loading doses of 120 mg on days 8 and 15 followed by ongoing treatment up to 12 months (27). Relapse of ABCs after denosumab discontinuation has been reported, but, notably, on restarting denosumab treatment, a similar response can be achieved again (29, 31, 32, 34).

#### Denosumab discontinuation

In the studies retrieved no patients were treated with zoledronate after denosumab discontinuation nor were there follow-up reports on the rebound phenomenon. In patients with ABCs the main concern after stopping denosumab is local recurrence of the lesion. Local lesion recurrence has been reported in 4 studies, with various dosage regimens: the “GCTB” treatment regimen for 2 years (33); the “GCTB” regimen for 2 years including surgical curettage of the lesion (31); the “GCTB” regimen for 1 month with subsequent monthly treatments of 60 mg for 3 months (31); and 60 mg monthly for 3 months followed by curettage (29). In all these reports there was no detail on using bisphosphonates after stopping denosumab. Of note, restarting denosumab treatment stopped lesions progression or reduced lesion size in all reported patients (29, 31, 34).

### Central giant cell granuloma of the jaw

#### Disease background

Central giant cell granuloma (CGCG) of the jaw (ICD10CM M27.1) is a rare bone lesion (36) that occurs more frequently in women than men, in the second or third decade, usually in the mandible (37, 38). CGCG may be mild or aggressive; in the latter case resulting in larger lesions and higher recurrence rates. Mandibular CGCG can cause pain, deformity, and tooth replacement with malocclusion (38, 39).

Lesions contain multinucleated giant OCs and a proliferation of fibroblasts (40). Because surgery, which requires en bloc resection or intralesional curettage, is associated with considerable morbidity, aesthetic consequences and a high rate of recurrence, it is logical to consider alternative therapies when symptomatic. Previous use of calcitonin (37) and glucocorticoids (41) has been reported, though with variable and modest results on pain and reduction in lesion size.

#### Treatment with denosumab

Denosumab has been associated with improvement in pain caused by CGCG and increased lesion mineralization (42, 43). In case reports (31 patients), denosumab has generally been given as a second-line treatment after previous therapy, such as corticosteroids or calcitonin, has failed, or as a first-line treatment, given at a dose of 120 mg monthly with loading doses at the beginning of treatment, similar to GCTB therapy. Recently, intralesional denosumab treatment has been reported with favorable outcomes (44, 45). No significant side effects have been reported in adults (43). Recurrence of disease has been reported, and patients may need long-term treatment (46-48). However, in aggressive forms, long-term treatment with a dose of 120 mg monthly may be needed, perhaps ultimately with a lower maintenance dose (49).

#### Denosumab discontinuation

In treating CGCG, asymptomatic mild self-limited hypercalcemia was reported in 2 cases after denosumab discontinuation (46). Additionally, local disease recurrence was noted in 4 out of 7 patients 1 year after discontinuing denosumab 120 mg, which was administered monthly followed by increase in dosing interval to every 2, 3, or 6 months with a median of 13 administered doses.

### Cherubism

#### Disease background

Cherubism (OMIM No. 118400) is a rare, autosomal dominant (AD) bone disease (AD in 94% of cases) (50), characterized by excessive bone degradation of the mandible and/or maxilla with fibro-osseous proliferative changes causing bone enlargement (9). There is pathogenic variant in the SH3 binding protein-2 *(SH3BP2)* gene (50), which encodes an adapter protein involved in adaptive and innate immune response signaling (51). AD disease but also sporadic manifestations due to either known or new pathogenic variants are observed (50). Cherubism manifests during childhood, and the disease progresses until puberty, in most cases with subsequent lesion regression afterward (9), with spontaneous regression reported only around puberty, although it may also persist or reappear during adulthood (52, 53). The term *“cherubism”* describes the clinical appearance of the patients, in whom a symmetrical enlargement of the maxilla and the mandible occurs. Other bones may occasionally be involved, such as the zygomatic bone (9). Pathological bone changes usually cause pain. The enlargement of the maxilla and/or mandible may lead to various complications (9), such as dental malalignment, visual impairment owing to lesions extending from the maxilla into the orbital region (54), respiratory impairment owing to airway obstruction (55), and impaired quality of life arising from the altered facial appearance and bone enlargement (9). Bone lesions in cherubism contain OCs-like giant cells (similar to those seen in giant cell tumor of bone) and fibroblasts (9, 50).

Treatment is usually conservative, as cherubism may regress spontaneously, although surgical intervention with curettage or resection and medical treatment with calcitonin have also been reported (9, 56).

#### Treatment with denosumab

The experience of denosumab use has recently been summarized (57). In Cailleaux's comprehensive summary (57), 6 of 18 (33%) patients with cherubism (mostly children) received denosumab 120 mg (following the GCTB regimen). Denosumab treatment, although off-label and heterogeneous in regimen and follow-up, was associated with radiographic sclerosis and suppression of lesion progression in several cases, but safety and long-term efficacy remain uncertain as not enough data were available. In adults, 2 cases have been reported. In one instance, documented improvement was noted at 6 months into a reduction in giant cells, bone formation, and bone sclerosis, without denosumab causing major side effects (58). A recent case report highlights the use of a different denosumab dosing regimen, in which 60 mg of denosumab once every 6 months for 2.5 years was associated with clinical improvement with reduced pain, improved functional outcomes, lesion size reduction, and bone formation, all of which prevented the need for surgery (59).

#### Denosumab discontinuation

Currently, there are no reports on denosumab discontinuation in adults.

### Fibrous dysplasia/McCune Albright syndrome

#### Disease background

FD/MAS (OMIM No. 174800) is a rare bone disease manifested by bone lesions caused by postzygotic *GNAS*-activating mutations (60). Lesions arise from formation of fibrous tissue within the bone. Fibrous lesions may be present within one bone (monostotic disease) or multiple bones (polyostotic disease). When FD is accompanied by extraskeletal manifestations, such as hyperfunctioning endocrinopathies, it is called McCune-Albright syndrome (61). The term *FD/MAS* encompasses the broad disease phenotype, from one monostotic lesion to extended polyostotic FD with multiple endocrinopathies, including the possibility of hyperfunctioning endocrinopathies development (62). Mutated osteoprogenitors in FD/MAS have an increased expression of RANKL with resulting increased osteoclastogenesis (2, 5). Patients may experience pain, deformities, fractures, and an impaired quality of life (63, 64).

There is no curative medical treatment for the disease, and currently intravenous bisphosphonates are widely used as symptomatic treatment (64).

#### Treatment of fibrous dysplasia with denosumab

In mouse models of FD/MAS, studies using denosumab showed that RANKL inhibition halts FD lesion progression and leads to increased mineralization (5). Use of denosumab in patients was first reported in 2012, with both clinical, namely pain reduction, and biochemical responses (65). Since then, multiple case reports, case series and a phase 2b clinical trial have been published (81 patients) concurring that denosumab is associated with decreased pain and overall improvement in bone biomarker responses in many patients (6, 66, 67). Furthermore, it is associated with decreased lesion activity as measured on NaF18 positron emission tomography/computed tomography (68), and even a reduction in lesion size (6, 66, 67). These effects have not been reported after treatment with bisphosphonates (69).

Most reported patients treated with denosumab had previously been refractory to bisphosphonate treatment (70-72), but treatment for naive patients was also included in some studies (71, 73, 74). Reasons for initiating denosumab treatment in these studies were either pain (6, 70, 71, 73) or to address lesion growth (66).

Treatment regimens differed: The first larger case series used 2 regimens (12 patients): a so-called “osteoporosis schedule”: with 6 monthly injections of 60 mg resulting in decreased turnover at month 3, but loss of effect (worsening of pain and bone turnover increase) at 6 months led to a change in treatment interval and an every 3 months schedule was introduced (70). This schedule was associated with persistent pain reduction and decrease of bone markers, which was observed in a larger study from the same group but other groups as well (66, 71, 72). However, some patients needed a higher dosage, of 120 mg, to achieve the desired effects (71, 75). Studies that reported on denosumab cessation effects showed that bone turnover returned to pretreatment levels (6), but mild rebound was observed, in some cases, accompanied by a mild asymptomatic hypercalcemia (71), although denosumab treatment duration was less than 3.5 years in these studies and therefore it might be of more concern with higher doses and longer treatment duration (6). Additionally, the risk of rebound may be higher in patients with extensive involvement of the skeleton or with high disease activity. Administrating zoledronic acid at discontinuation of denosumab attenuated hypercalcemia in adult patients with FD/MAS (6, 65).

Denosumab was well tolerated, with medication-related reported side effects during denosumab treatment in FD/MAS including secondary hyperparathyroidism, asymptomatic hypocalcemia and asymptomatic hypophosphatemia, oral blisters, and one case of osteonecrosis of the jaw, located in an FD lesion, in a patient who already had been on long-term high-dose bisphosphonates (71).

Denosumab is a promising treatment for adult patients with FD/MAS but its long-term effect on pain and optimum dosing schedule still need to be elucidated. It seems reasonable to initiate treatment with 60 to 120 mg followed by an every 3 months schedule until sufficient pain reduction is achieved in patients not able to participate in ongoing clinical trials. After pain reduction has been achieved, treatment can be followed by bisphosphonate treatment at discontinuation to temper a potential rebound phenomenon. At the moment, a randomized double-blind placebo-controlled trial (NCT05966064) aims to investigate whether every 3 months 120-mg denosumab will improve the clinical, radiological, and biochemical manifestations of FD bone lesions in adults, with pain reduction as the primary end point. A recent study by Palmisano and colleagues (76) in a FD/MAS mouse model compared different treatments (zoledronic acid/short term anti-RANKL with discontinuation/long-term anti-RANKL/short-term anti-RANKL with zoledronic acid one shot during anti-RANKL treatment and one shot after the last treatment). There it was seen that anti-RANKL treatment rapidly reduced bone turnover and increased the bone mass of affected skeletal sites. However, FD lesions recurred shortly after discontinuation when no follow-up treatment was given. The beneficial effects of anti-RANKL were preserved by the addition of zoledronic acid. The results of this study imply that the addition of zoledronic acid to denosumab might be a useful approach to treat FD/MAS, but this remains to be investigated in humans.

#### Denosumab discontinuation

The effect of stopping denosumab in a bone lesion characterized by high bone turnover is best observed in FD/MAS. In children, bone turnover rebound is more vigorous, and more commonly associated with clinically significant hypercalcemia. In pediatric FD patients, both hypercalcemia and rapid lesional growth after denosumab discontinuation have been reported (11, 65), while in adults these adverse effects tend to arise on withdrawal from high drug doses and where there is extensive skeletal disease (6, 71, 77). In contrast to children, lesional growth after denosumab discontinuation has not been described in adults with FD. This may relate to the relative stability of FD lesions after skeletal maturity due to reduction of the number of GNAS-mutated immature osteoblasts (65, 78). Severe hypercalcemia was reported after denosumab discontinuation, 8 weeks after zoledronate, in a patient with high skeletal burden and high bone turnover undergoing treatment with high doses of denosumab in a phase 2b clinical trial, which resolved after receiving treatment with fluids and antiresorptives (6). In adults, mild asymptomatic rebound hypercalcemia responds to intravenous zoledronate (71, 77). Among reported studies, zoledronate acid has been administered at discontinuation at different time points 4 weeks or 6 months after the last denosumab injection, or 3 months after the last injection due to rebound hypercalcemia (6, 66, 71). To our knowledge, there have been no reports of incident vertebral fractures occurring after denosumab discontinuation in FD/MAS.

### Gorham-Stout disease

#### Disease background

GSD (OMIM No. 123880) is a rare bone disease characterized by increased bone resorption and diffuse proliferation of lymphatic vessels (79). GSD occurs mostly in children or young adults, usually in the third decade (80). Potentially all bones can be affected, but GSD most commonly affects maxillofacial bones (81). Etiology is not precisely defined, and different theories have been proposed on what the primary pathology might include: increased OC activity with osteolysis, angiogenesis/lymphangiogenesis, and decreased osteoblast activity (80). As OCs may not be seen on tissue specimens, it is presumed that osteolysis occurs as a result of OC precursors being sensitive to humoral factors leading to increased osteoclastogenesis (82).

Patients with GSD may experience pain, deformity of affected bones, and functional impairment, but there is also an increased risk of fracture at the lesion site (80). Some GSD locations, such as the spine or skull, may lead to secondary localized and debilitating complications such as neurologic deficits or cerebrospinal fluid (80). Such effects can be fatal (83). Established treatments for GSD include radiotherapy and angiogenesis inhibitors (80). Bisphosphonates have been used to reduce OC activity, with positive results on reducing disease progression, but this is not found in all patients (80).

#### Treatment with denosumab

Given that OC activity is dependent on RANKL activity in GSD, and may be key to disease pathogenesis, denosumab may provide a potentially useful treatment in targeting the osteoclastogenesis, as reported in 3 cases. Indeed, 60 mg every 6 months has been associated with symptom improvement and slow disease progression (84, 85). More recently, Zhang and colleagues (86) reported that a combination of 1 dose of denosumab 60 mg with teriparatide 20 μg daily (to enhance osteogenesis) for 2 months was associated with clinical improvement in a patient with oral bisphosphonate–refractory GSD. The patient experienced pain relief, lesion size reduction, and evidence of bone formation, without notable side effects.

#### Denosumab discontinuation

Currently, there are no reports on denosumab discontinuation in adults.

### Hajdu-Cheney syndrome

#### Disease background

HCS (OMIM No. 102500) is a rare, AD, multisystemic disease affecting the skeleton, cardiovascular, and neurological system. Specific phenotypic features occur particularly craniofacial anomalies (hypertelorism, bushy eyebrows, micrognathia, small mouth with dental anomalies, low-set ears, and short neck), which may change as the patient gets older (87, 88). In adults, skeletal manifestations are due to bone loss represented by acro-osteolysis in the distal phalanges, severe osteoporosis with increased risk of vertebral fractures, but also scoliosis, kyphosis, and pulmonary complications (87, 88). HCS is associated with gain-of-function variants in the *NOTCH2* gene. The *NOTCH* pathway is involved in RANK modulation, and *NOTCH2* pathogenic variants lead to increased osteoclastogenesis (87, 89).

Bisphosphonates have been used to address the bone loss of disease-associated osteoporosis and acro-osteolysis, with good results for osteoporosis, with increased vertebral bone density, but not for the acro-osteolysis (87, 89).

#### Treatment with denosumab

Denosumab treatment might be expected to be successful on the basis that gain-of-function variants in *NOTCH2* stimulate RANKL-dependent osteoclastogenesis (89). Accordingly, the first report of denosumab treatment in adults used 60 mg every 6 months, which improved vertebral bone density, but without affecting acro-osteolysis, which progressed despite denosumab (90). Another report of a patient treated with the same dosing regimen emphasized stabilization/nonprogression of the acro-osteolysis (91). Potentially, it could be hypothesized that a possible explanation for the absence of an effect on acro-osteolysis might be due to an “underdosing,” as many patients with RBDs need higher doses of denosumab due to their high turnover compared with osteoporosis patients; however, as yet there have been no reports to substantiate this.

#### Denosumab discontinuation

Currently, there are no reports on denosumab discontinuation in adults.

### Langerhans cell histiocytosis

#### Disease background

LCH (OMIM No. 603672) is a rare disease, caused by clonal proliferation and dissemination of Langerhans-like cells. LCH may affect one or multiple organs and has been recently recognized as a hematological neoplastic disease; it is included in the “Langerhans group” (L-group) of the histiocytic disorders, with a somatic p.V600E pathogenic variant in *BRAF* being found in around half of all cases, while up to 20% of patients may harbor other pathogenic variants in the *MAPK* pathway (92). In the skeleton, LCH manifests as lytic lesions and loss of bone mass (8, 93). LCH is more often diagnosed in children than adults, and disease pathophysiology is not completely known. However, the presence of giant OCs and RANKL upregulation in diverse lesions with concomitant activation of *p65 NFĸB* and compensatory increase in circulating OPG may also play a role in disease pathogenesis (8, 94). In adults, the mandible, skull, and long bones are the most commonly affected skeletal sites. Typical effects of skeletal lesions are pain and deformity (8).

#### Treatment with denosumab

The first report of denosumab use (in 2 LCH adult female patients) showed that 4 doses of denosumab 120 mg administered every 2 months was associated with pain relief after even the first dose and a reduction in the size of lesions, without causing side effects (95). More recently, a phase 2b clinical trial (10 patients) (96) showed that 4 administrations of denosumab 120 mg every 2 months were effective in adult LCH patients with mild skeletal symptoms. The treatment achieved an 80% overall response in various tissue involvement besides bone and was very well tolerated, without causing major side effects. As courses of denosumab treatment were given only briefly, no bisphosphonate consolidation treatment was given. However, patients did not exhibit a rebound increase in bone turnover nor bone mineral density loss or other skeletal events associated with denosumab discontinuation (97).

### Other diseases

There have been isolated reports of denosumab use reducing bone pain in trichorhinophalangeal dysplasia type II (also known as Langer-Giedion syndrome; OMIM No. 150230) (98) and pseudomyogenic hemangioendothelioma of bone (99). Despite being primarily a disease of dysfunctional osteoblasts in melorheostosis, the RANK-RANKL pathway might be affected as well, perhaps due to the coupling of formation and resorption. In a single case report, the clinical course of a 20-year-old woman with melorheostosis improved after treatment with denosumab (100). Denosumab was also associated with pain reduction in a 71-year-old patient with diffuse sclerosing osteomyelitis (101), and a 50-year-old patient with chronic nonbacterial osteomyelitis (102). Potentially, targeting the RANK-RANKL pathway might also be beneficial for diseases caused by *TNFRSF11A* mutations with subsequent increased RANK-RANKL activity. However, to date, no case reports for denosumab treatment have been published.

### Considerations

There is a lack of data and information on denosumab discontinuation in treating RBDs. Nevertheless, besides in FD/MAS, in none of the aforementioned diseases has an exit strategy been described, which may have an essential role in tackling the risk of relapse.

We note for example most of the cases reported were of aggressive rather than mild disease, in which the risk of rebound high bone turnover after denosumab discontinuation is higher. None of the studies used bisphosphonates as an exit strategy. In all reports of adult disease, we note a paucity of reported side effects of denosumab or otherwise the risk of local recurrence. Guidance on maintenance dosing, either by decreasing the dose or increasing the time interval, would be valuable. However, given the limited and heterogeneous data available, and particularly the reliance on case reports and small case series, there is insufficient evidence to support specific recommendations on maintenance regimens or interval extension strategies. However, in most reports the maintenance dose has been defined as the lowest dose possible while remaining good clinical results.

In all the RBDs we have discussed here, it would seem logical, and there is a need to know in a more granular way, that disease/lesion recurrence occurs when denosumab is discontinued. For example, lesion pain, lesion growth/regrowth, and bone turnover (bone resorption) may all have clinical relevance and correlation with an adverse effect of denosumab discontinuation. It is important to consider that reports of pain reduction in case reports are inherently subjective and often unreliable, and that robust evaluation of pain outcomes requires placebo-controlled clinical trials. However, conducting such trials in rare diseases is challenging due to the limited number of eligible patients. Data collected through rare bone disorder registries may provide valuable information to support high-quality research (Supplemental material 5) (21).

Effects may have relevance to the denosumab dosing regimen used, and to the drug's benefit: risk profile in treating a specific RBD. Moreover, there is a need to study the abrogating effects of zoledronate on rebound high bone turnover after denosumab discontinuation, similar to osteoporosis treatment protocols.

In general, denosumab treatment in adults is well tolerated (103). In RANKL-mediated benign diseases, this is not different, even though higher doses are used. Reported side effects are similar to those observed in patients treated with denosumab for osteoporosis, but can be more pronounced in case of very high turnover (eg, more severe hypophosphatemia or hypercalcemia) after discontinuation. Furthermore, medication-related osteonecrosis of the jaw and atypical femoral fractures have been reported as well (6, 17, 71). Most RBD patients treated with denosumab in childhood lack standardized transition protocols to adult care, an unmet need that must be addressed to prevent treatment gaps and complications (104).

**Figure 2:**
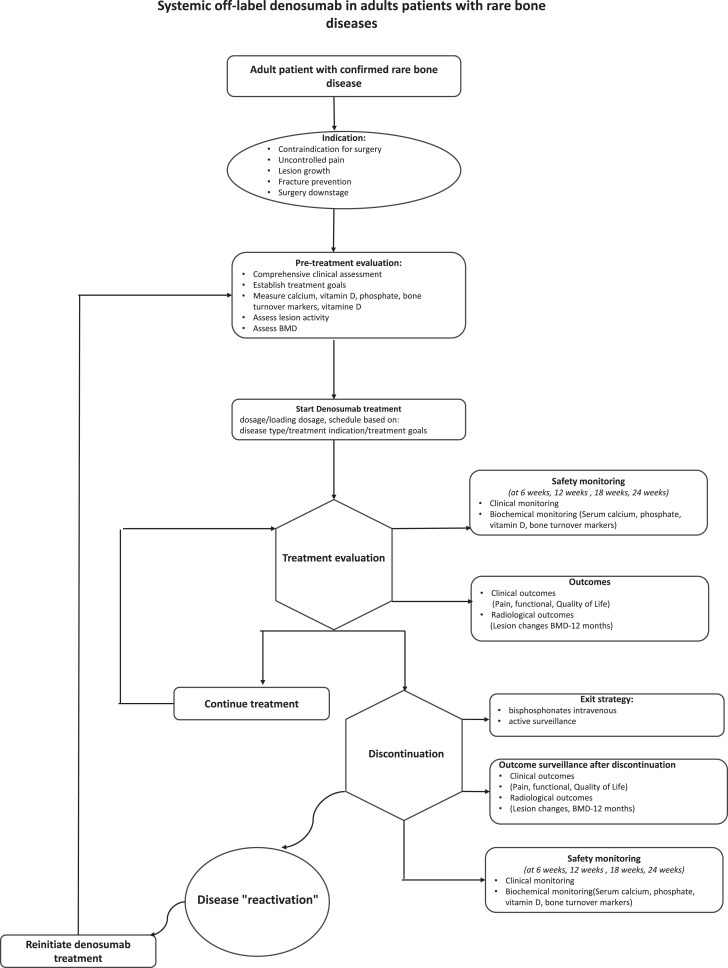
Systemic off-label denosumab in adult patients with rare bone diseases flowchart.

In many diseases, treatment initiation is associated with a frequent dosing interval, although there is no clear guidance on when to proceed to a “maintenance” schedule with longer intervals. Although this step can be guided by clinical aspects such as reduction in pain or stabilization of the disease, serial bone mineral density measurements might be beneficial to assess the effect of denosumab on healthy bone and aid the adaption of the treatment schedule. Bone density improvement has been reported in patients with Hajdu-Cheney disease treated with denosumab (90, 91), while in other diseases the focus during follow-up has been on target lesions and not on healthy bone. We propose an algorithm for systemic denosumab use in RBDs (Fig. 2).

### Conclusion

Denosumab has been used for different indications in the management of adults RBDs, aiming to reduce pain, as well as potential lesion reduction and stopping disease progress.

Denosumab appears to be a viable and feasible treatment for a range of RBDs, successfully improving the clinical picture with reduced pain. In some cases, it has been associated with stopped or amended lesion progression, and increased lesion mineralization has also been reported. However, to date, there is no consensus on an optimum dosing regimen, optimum timing of treatment (eg, in relation to surgery), or on the desired goals (lesion pain, growth, surgery downstage). Also, long-term follow-up data regarding denosumab discontinuation are missing as most studies cover only 1 year. Therefore, considering the current limited evidence and possible complications, treatment with denosumab for adults with RBDs should be carried out in collaboration with an expert center where a thorough assessment by a multidisciplinary team should be conducted, not only including denosumab treatment indication, but also to set treatment goals alongside treatment monitoring with objective measures like imaging or patient-reported outcome measures like pain assessment (visual analog scale or Brief Pain Inventory) or function. Furthermore, we advocate better monitoring of patients stopping denosumab. Considering the rarity of many diseases, large randomized clinical trials are not to be expected, but currently trials are ongoing in FD/MAS (NCT05966064; NCT03571191) and giant cell tumor of bone (NCT04586660; NCT03358212). Therefore, the summary of evidence now lies within multiple case reports and observational studies that include different schedules of treatment, different follow-up, and different information on long-term use or discontinuation. This highlights the crucial role of RBD registries in collecting data in a standardized manner to lead to pertinent conclusions for future recommendations (105, 106). Such registries have been established in Europe to collect standardized data on RBDs, enhance understanding of their natural history, and enable accurate statistical analysis (105, 106). Finally, fully unlocking this potential treatment will be of tremendous benefit for patients with RBDs in whom no treatment options are available, and research toward treatments is rare and poorly funded considering the low number of patients. The use of denosumab in additional conditions deserves further investigation, as it already has been associated with considerable improvements in certain patient populations.

## Data Availability

Data sharing is not applicable to this article as no datasets were generated or analyzed during the current study.

## Abbreviations

ABCS: aneurysmal bone cysts
AD: autosomal dominant
CGCG: central giant cell granuloma
ECTS: European Calcified Tissue Society
FD/MAS: fibrous dysplasia/McCune-Albright syndrome
GCTB: giant cell tumor of the bone
GSD: Gorham-Stout disease
HCS: Hajdu-Cheney syndrome
LCH: Langerhans cell histiocytosis
OCs: osteoclasts
OPG: osteoprotegerin
RBDs: rare bone diseases
